# Heterotic Patterns of Temperate and Tropical Maize by Ear Photometry

**DOI:** 10.3389/fpls.2021.616975

**Published:** 2021-06-14

**Authors:** Seth A. Tolley, Amritpal Singh, Mitchell R. Tuinstra

**Affiliations:** ^1^Department of Agronomy, Purdue University, West Lafayette, IN, United States; ^2^Advanta Seeds, College Station, TX, United States

**Keywords:** ear photometry, heterotic groups, hybrid breeding, multivariate analysis, tropical maize, high-throughput phenotyping

## Abstract

As the plant variety protection (PVP) of commercial inbred lines expire, public breeding programs gain a wealth of genetic materials that have undergone many years of intense selection; however, the value of these inbred lines is only fully realized when they have been well characterized and are used in hybrid combinations. Additionally, while yield is the primary trait by which hybrids are evaluated, new phenotyping technologies, such as ear photometry (EP), may provide an assessment of yield components that can be scaled to breeding programs. The objective of this experiment was to use EP to describe the testcross performance of inbred lines from temperate and tropical origins. We evaluated the performance of 298 public and ex-PVP inbred lines and 274 Drought Tolerant Maize for Africa (DTMA) inbred lines when crossed to Iodent (PHP02) and/or Stiff Stalk (2FACC) testers for 25 yield-related traits. Kernel weight, kernels per ear, and grain yield predicted by EP were correlated with their reference traits with *r* = 0.49, *r* = 0.88, and *r* = 0.75, respectively. The testcross performance of each maize inbred line was tester dependent. When lines were crossed to a tester within the heterotic group, many yield components related to ear size and kernels per ear were significantly reduced, but kernel size was rarely impacted. Thus, the effect of heterosis was more noticeable on traits that increased kernels per ear rather than kernel size. Hybrids of DTMA inbred lines crossed to PHP02 exhibited phenotypes similar to testcrosses of Stiff Stalk and Non-Stiff Stalk heterotic groups for yield due to significant increases in kernel size to compensate for a reduction in kernels per ear. Kernels per ear and ear length were correlated (*r* = 0.89 and *r* = 0.84, respectively) with and more heritable than yield, suggesting these traits could be useful for inbred selection.

## Introduction

Maize production in the United States totaled 363.2 billion kg on 33.5 million hectares with an average yield of 10,860 kg ha^–1^ in the five growing seasons from 2015 to 2019; an incredible feat considering the nationwide average yield of 2,950 kg ha^–1^ in 1956, which was the beginning of the single-cross hybrid maize era (USDA NASS). In the early 20th century, maize was commonly grown as an open-pollinated variety and yield improvement was stagnant. Since the implementation of double-cross and subsequently single-cross hybrids, maize yields have increased at a rate of 48 and 119 kg ha^–1^, respectively (USDA NASS). Duvick (2005) attributes about 50% of the increases in maize yields to hybrid maize breeding and the development of superior genetics, while optimized genotype placement and management practices are other key factors.

In 1970, the Plant Variety Protection Act (PVP) allowed commercial breeding programs to register varieties and inbred lines as intellectual property restricting the use, sale, and importation of this material for 20 years (Beckett et al., 2017). Inbred lines selected in commercial breeding programs are highly advanced and have undergone many rounds of intense selection. As PVPs expire, public breeding programs have access to these highly advanced inbred lines that can be used to quickly incorporate useful alleles into breeding programs. Many studies have used genomic and pedigree information to characterize ex-PVP and founder temperate inbred lines (Mikel, 2006, 2008, 2011; Mikel and Dudley, 2006; Nelson et al., 2008; White et al., 2020); however, phenotypic information describing these temperate inbred lines is less common.

Commercial maize is grown as a hybrid F_1_ cross of inbred lines from divergent heterotic groups to leverage heterosis (Shull, 1908, 1909a, 1909b, 1911, 1914; East, 1909, 1936). In the United States, maize germplasm is largely classified into three predominant heterotic groups: Stiff Stalk (SS), Non-Stiff Stalk (NS), and Iodent (IO) (Nelson et al., 2008; Beckett et al., 2017; White et al., 2020). Maize breeders exploit the heterotic pattern between these complementary heterotic groups to form hybrids with greater yield potential than their inbred parents. Inbred lines within heterotic groups have been characterized and selected for their combining ability with inbred lines from other heterotic groups. They are evaluated for their testcross rather than *per se* performance (Bernardo, 2014). To conserve the genetic diversity between heterotic groups, germplasm improvement within heterotic groups is typically limited to recycling and recombining the best inbred lines in a population through reciprocal recurrent selection (Duvick et al., 2004; Mikel, 2008). While the founding germplasm for the United States Corn Belt Dent population was large and diverse (Duvick et al., 2004), Mikel and Dudley (2006) stated that much of the current commercial germplasm can be traced back to seven progenitor inbred lines: B73, LH82, LH123, PH207, PH595, PHG39, and Mo17. Yield stagnation due to limited genetic diversity has not been evident, though researchers question whether this bottleneck could restrict future genetic gains (Holland and Goodman, 1995; Goodman, 2005; Nelson and Goodman, 2008). Incorporation of tropical germplasm is one solution to broadening genetic diversity and is a goal of many breeding programs (Uhr and Goodman, 1995a,b; Duvick et al., 2004; Henry et al., 2014). Thus, understanding the combining ability of temperate inbred lines with tropical germplasm could be an important consideration for public and private breeding programs to sustain genetic improvement of maize in the 21st century.

The International Maize and Wheat Improvement Center, CIMMYT, hybrid maize breeding program began in 1985 (Vasal et al., 1992). As a large collection of germplasm was available, the first goal of the program was to assess combining ability and empirically determine heterotic groups. Heterotic groups explored included Tuxpeño, Cuban flints, Coastal tropical flints, ETO, Tuson, Chandelle, Haitian yellow, and Perla (Vasal et al., 1999). However, concurrent development of several heterotic groups for their combining ability was difficult and was further simplified as Tuxpeño, heterotic group A (dent kernel type), and non-Tuxpeño, heterotic group B (flint kernel type) (Vasal et al., 1999; Wu et al., 2016; Cupertino-Rodrigues et al., 2020). Nevertheless, due to the relatively short-term selection of the heterotic groups, tester dependent heterotic group classification of a given line, and diversity in the base population, heterotic groups A and B can often be difficult to classify (Wu et al., 2016).

The recent emergence of high-throughput phenotyping is an important tool with the potential to relieve the bottleneck of testing programs (Furbank and Tester, 2011; Araus and Cairns, 2014; Araus et al., 2018). High-throughput phenotyping can increase the genetic gain by increasing selection intensity, phenotype repeatability, and trait heritability (Araus et al., 2018). Selection intensity is a function of the number of lines selected compared to the number of lines evaluated. With high-throughput phenotyping, larger populations can be evaluated and more stringent selection intensities can be imposed. Response to selection can be increased by minimizing the non-genetic variance by increasing phenotype repeatability and trait heritability (Bernardo, 2014). Minimizing non-genetic variance is often the case of appropriate experiment design and statistics involving blocking, randomization, and replication. High-throughput phenotyping allows for greater replication and reduced between-measurement error by removing the human-element of phenotyping. Limitations of low-throughput phenotyping methods such as its time-consuming and laborious nature, often force breeding programs to limit selection to yield evaluated in multi-environment, multi-year trials; even though the heritability of yield is among the lowest of commonly evaluated traits (Furbank and Tester, 2011). Selection based on yield components that are highly related to and more heritable than yield could be beneficial.

Phenotyping of maize yield components such as ear and kernel properties has been one of the interests for breeding programs and research geneticists. Typical yield components include kernel number, kernel weight, and ears per plant. Previous studies have used manual methods to record ear and kernel traits to dissect the genetic basis of these traits (Flint-Garcia et al., 2005; Ross et al., 2006). However, the process is time-consuming, labor-intensive and difficult to scale to large breeding programs (Cooper et al., 2014). Using a high-throughput phenotyping method known as ear photometry (EP), yield components, such as ear and kernel characteristics, can be measured or predicted (Grift et al., 2017; Miller et al., 2017; Makanza et al., 2018). These studies reported great prediction accuracies for many yield-related traits, giving credibility to the idea that EP could be valuable in assessing large breeding populations.

In 2017 and 2018, testcross hybrids of ex-PVP inbred lines from the United States and tropical inbred lines from the drought tolerant maize for Africa (DTMA) panel from CIMMYT were assessed for 25 yield-related traits when crossed to SS and/or IO testers. Representative ears were hand harvested from each plot for EP studies. The objectives of this experiment were to (1) validate the use of ear photometry on a diverse set of hybrids representing temperate and tropical maize, (2) characterize the relationships among and heritability of ear photometry traits, and (3) describe the heterotic patterns of temperate and tropical inbred lines in testcross hybrids using ear photometry.

## Materials and Methods

### Experimental Design and Germplasm

This experiment was grown in the summer of 2017 and 2018 at the Purdue University Agronomy Center for Research and Education (40°48′ N, 86°99′ W). The soil type at this location is a Chalmers silty clay loam (Fine-silty, mixed, superactive, mesic Typic Endoaquolls) (Purdue Agriculture Data Engine). Experimental plots followed a maize-soybean crop rotation with planting dates of June 1, 2017 and April 30, 2018. In both years, the fields were cultivated prior to planting. Fertilizer was applied in the fall of 2016 as 168 kg ha^–1^ of mono-ammonium phosphate (11-52-0) and 224 kg ha^–1^ of potash (0-0-60), and in the spring of 2017, 180 kg ha^–1^ of pre-plant ammonium nitrate was applied. Weed control [Bicep II (Atrazine + S-metolachlor), Syngenta] was applied pre-plant at a rate of 3.7 kg ha^–1^. In 2018, nitrogen was applied in mid-March at a rate of 224 kg ha^–1^ of ammonium nitrate. Weed control [Bicep II (Atrazine + S-metolachlor), Syngenta], was applied pre-plant at a rate of 4.7 kg ha^–1^. Additionally, [Laudis (Tembotrione), Bayer], was applied after planting at a rate of 210 g ha^–1^. Prior to silk emergence in 2018, insecticide [Sevin (Carbaryl), Bayer] was applied due to a Japanese Beetle infestation.

Experimental plots were two rows 4.5 m long by 1.5 m wide with a spacing of 76 cm in 2017 and 3 m long by 1.5 m wide with a spacing of 76 cm in 2018 planted to a population of 74,000 seeds ha^–1^ in each year. The hybrids were blocked by temperate or tropical origin and tester to promote pollination among hybrid types and evaluated in a randomized complete block design with two replications.

Germplasm consisted of temperate inbred lines from public breeding programs and available ex-PVP inbred lines and DTMA inbred lines from CIMMYT. Testcross performance of 286 temperate inbred lines and the 274 DTMA inbred lines were evaluated in combination with PHP02 (IO). The choice of a commercial IO tester for the tropical germplasm differentiates this study from previous work which tested *per se* performance (Uhr and Goodman, 1995b) and combining ability to B73/Mo17 (Uhr and Goodman, 1995a). Additionally, 298 temperate inbred lines were evaluated for testcross performance to 2FACC (SS). Heterotic group assignment (SS, IO, or NS) of temperate inbred lines used in this study were previously described by Beckett et al. (2017). CIMMYT provided the heterotic group classification (A, B, or AB) of the DTMA inbred lines.

### Ear Photometry Pipeline

Prior to machine harvest, 10 representative ears were selected from the plots in 2017 and 5 representative ears were selected from the plots in 2018 and dried to about 15% moisture followed by red-green-blue (RGB) imaging. These ears were imaged from a single angle using a Canon EOS Rebel T6i camera and an imaging system from Corteva Agriscience formerly DuPont Pioneer (Hausmann et al., 2009). Before imaging, the ears were thoroughly cleaned to remove silks and other debris. In total, more than 4,200 RGB images were acquired and processed to determine the phenotypes of more than 21,000 ears.

Common image processing techniques such as filtering, thresholding, edge finding, edge enhancement, color selection, spectral filtering, and water shedding were used to process images in a semi-automated process (Hausmann et al., 2009). Using a supervised classification method, ears (with kernels) and cobs (without kernels) were extracted from the background. As a template, a representative image with variation in kernel color was selected from each year to define the pixel attributes associated with the cob and kernels. Length measurements were calibrated using a reference image of a ruler.

EP was used to measure or predict 25 traits to provide unique insight into the characteristics of an ear. Traits measured or predicted in EP include, but are not limited to, grain yield (PHTYLD), kernels per ear (PHTKPE), average single kernel weight (KERWGT), and ear length (EARLGT). For a full description and heritability of the 25 EP traits, please refer to Table 1: Ear Photometry.

**TABLE 1:** Description of the phenotypes evaluated in this study.

**Trait Sections**	**Traits**	**Units**	**Heritability**		**Definition**
			**Entry-Mean**	**Plot-Mean**	
Reference Physiology	AD	GDD*	0.91	0.75	Growing degree days to reach anthesis
	SD	GDD	0.92	0.78	Growing degree days to reach silking
	ASI	GDD	0.51	0.22	Anthesis-silking interval measured in growing degree days
	ASI	Days	0.60	0.31	Anthesis-silking interval measured in days
	PH	Cm	0.91	0.83	Height from ground level to the top collared leaf
	EH	Cm	0.86	0.76	Height from ground level to the node of the primary ear
Reference Yield Components	REFKW	g kernel^–1^	0.79	0.66	Reference average kernel weight manually measured
	REFKPE	count ear^–1^	0.84	0.73	Reference kernels per ear manually measured
	REFYLD	g ear^–1^	0.82	0.70	Reference grain yield manually measured
	REFYLD18‡	kg ha^–1^	0.69	0.52	Reference grain yield at 15% moisture measured by combine
	MOISTURE‡	%	0.80	0.66	% moisture at harvest measured by combine
Ear Photometry	PHTYLD	g ear^–1^	0.52	0.25	Grain yield
	PHTKPE	count ear^–1^	0.60	0.30	Total number of kernels per ear
	KERWGT	g kernel^–1^	0.51	0.25	Average kernel weight
	EARAREA	cm^2^ ear^–1^	0.61	0.32	Ear area
	EARBOX	–	0.49	0.22	Ear boxiness
	EARCW	–	0.65	0.38	Ear central width
	EARLGT	cm ear^–1^	0.71	0.41	Total length of cob
	EARPER	cm ear^–1^	0.70	0.40	Ear perimeter
	EARTR	cm^2^ ear^–1^	0.46	0.19	Ear tip ratio
	EARVOL	cm^2^ ear^–1^	0.56	0.28	Ear volume
	EARWTH	cm ear^–1^	0.63	0.35	Width of ear including kernels and cob
	ETB	–	0.50	0.22	Ear tip boxiness
	KERARE	cm^2^ kernel^–1^	0.67	0.39	Average area per kernel
	KERCC	–	0.54	0.25	Kernel central count
	KERFIL	cm^2^ (cm^2^)^–1^	0.50	0.25	Percent of total ear area with filled kernels
	KERLEN	cm kernel^–1^	0.58	0.30	Average kernel length
	KERMAXD	cm kernel^–1^	0.59	0.30	Kernel maximum diameter
	KERMEAND	cm kernel^–1^	0.71	0.44	Kernel mean diameter
	KERMIND	cm kernel^–1^	0.74	0.46	Kernel minimum diameter
	KERPER	cm kernel^–1^	0.75	0.47	Kernel perimeter
	KERWTH	cm kernel^–1^	0.73	0.45	Average kernel width
	PHTKR	count ear^–1^	0.63	0.32	Kernel row number
	PHTKPR	count ear^–1^	0.64	0.34	Total number of kernels per row
	SCTTER	cm^2^ (cm^2^)^–1^	0.51	0.26	Percent of ear area lost due to scatter grain
	TKERAB	cm cm^–1^	0.42	0.17	Percent of ear length affected by kernel abortion

Trait heritability on an entry-mean and plot-mean basis using Equations 2, 3. Traits in the reference section refer to traits measured manually or by combine, while traits in the ear photometry section were measured using the ear photometry platform. *Daily Growing Degree Days (C°) = $\frac{T_{\text{max}}\,C-T_{\text{min}}\,C}{2}-10\,\text{C}$ If Max Temp > 30°C, then Max Temp = 30°C. If Max Temp < 10°C, then Max Temp = 10°C. If Min Temp > 30°C, then Min Temp = 30°C. If Min Temp < 10°C, then Min Temp = 10°C. ^†^Only measured in 2017. For these traits, the effect of Year was removed from the model. ^‡^Only measured in 2018. For these traits, the effect of Year was removed from the model.

### Reference Trait Measurements

Reference traits were measured either to provide additional in-season information related to a hybrid or to validate the EP platform. Reference traits were measured throughout the growing season. The description and heritability of the reference traits are provided in Table 1: Reference Physiology. Anthesis date (AD) and silking date (SD) were recorded as date when 50% of the plot reached anthesis or silking, respectively. Growing degree days (GDD) (C°) was calculated for each day from planting to AD and SD and summed to determine accumulated GDD. GDD for each day was calculated using the formula, GDD = [(T_max_ + T_min_)/2] − 10. When the maximum and minimum temperatures were greater than 30°C or less than 10°C, then T_max_ and T_min_ were set to 30 and 10°C, respectively (Gilmore and Rogers, 1958). Anthesis-to-silking interval (ASI) was the duration between AD to SD and was measured in GDD and days. In 2017, plant height (PH) was measured from the ground to the top collared leaf, and ear height (EH) was measured from the ground to the node of the primary ear.

The accuracy of EP predicted traits PHTYLD, PHTKPE, and KERWGT was assessed through manual measurements of the reference traits. The abbreviations for reference traits start with *REF* and the heritabilities and descriptions of these traits are provided in Table 1: Reference Yield Components. In 2017, the 10 ears per plot used in EP were shelled (Agriculex Single Ear Corn Sheller). The kernels of the 10 ears were combined and a seed counter (VMek Sorting Technology) was used to measure the number of kernels. The total weight of the kernels was measured using an Ohaus (NVT10001/1, Ohaus Corporation, Parsippany, NJ, United States) balance. Kernel number and total weight were subsequently divided by 10 to get measurements on a per ear basis. Kernel number per ear (REFKPE) manually measured was the reference trait for PHTKPE. Total weight per ear (REFYLD) manually measured was the reference trait for PHTYLD. Reference kernel weight (REFKW) was the quotient of REFYLD (g ear^–1^) divided by REFKPE (count ear^–1^) and was used as the reference trait for KERWGT. The average EP predicted PHTYLD, PHTKPE, and KERWGT was correlated to their corresponding reference measurement. Subsequently, this validation dataset was split into temperate (*n* = 989) and tropical (*n* = 424) origin to verify the use of EP in these perspective backgrounds and are shown in Supplementary Figure 1.

In addition to using reference yield (g ear^–1^) to validate the EP platform, reference yield (REFYLD18) (kg ha^–1^) was measured on a per plot basis (*n* = 1,568) in 2018 using a plot combine (Kincaid 8-XP, Haven, KS, United States) with grain weights standardized to 15% moisture. Stand count was used as a covariate in the linear model between REFYLD18 and PHTYLD to limit the variability in yield due to differences in stand. Moisture was measured as the percent moisture in the grain at harvest with the plot combine. This validation dataset was split into temperate (*n* = 1,120) and tropical (*n* = 448) origin to verify the use of EP in these perspective backgrounds (Supplementary Figure 1).

### Genotypic Data

Genotypic data for the temperate ex-PVP and public breeding lines was provided by the Rocheford Lab of Purdue University and previously described in Beckett et al. (2017). Briefly, the genotypic information of 291 temperate lines was aligned and merged with 58 additional ex-PVP inbred lines. In total, there were 1,281,671 Single Nucleotide Polymorphisms (SNPs) in the merged genotype file for the 349 temperate inbred lines.

Genotypic data for the DTMA germplasm was sourced by CIMMYT from an online repository^1^. This dataset consisted 955,690 SNPs for 282 inbred lines and have been previously described by Guo et al. (2020), Yuan et al. (2019), and Wu et al. (2016). Once aligned and merged, the dataset of temperate and tropical germplasm consisted of 533 inbred lines phenotyped in this project with 755,339 common SNPs.

### Statistical Analysis

Data analyses were performed in R (R Core Team, 2019). Best linear unbiased predictions (BLUPs) were predicted using lme4::lmer (Bates et al., 2015) according to equation 1. While the Hybrid × Year interaction was significant for most traits, the amount of variation explained by the Hybrid was typically far greater than the interaction. As such, BLUPs were predicted over the 2 years rather than BLUPs for each year using the following equation.

(1) $$Y_{\text{ijkl}}=\mu +H_{i}+Y\,r_{j}+H\,Y\,r_{i\,j}+R\,{\left(Y\,r\right)}_{j\,k}+\varepsilon_{\text{ijkl}}$$

Where Y*_ijkl_* is the phenotypic measurement of the *i*^th^ hybrid, in the *j*^th^ year, in the *k*^th^ rep. μ represents the grand mean; *H*_i_ is the random effect of the *i*^th^ hybrid; *Yr*_j_ is the fixed effect of the *j*^th^ year; *HYr*_ij_ is the random interaction effect of the *i*^th^ hybrid in the *j*^th^ year; *R(Yr)_jk_* is the fixed effect of the *k*^th^ rep nested in the *j*^th^ year. ε is the random residual error term associated with each phenotypic measurement. Significant differences of heterotic groups were determined using analysis of variance (ANOVA) of the BLUPs. When ANOVA was significant, Tukey test was performed to distinguish between the heterotic groups in R library agricolae::HSD.test.

Broad-sense heritability (Nyquist and Baker, 1991; Piepho and Möhring, 2007) using variance components estimated through restricted maximum likelihood (REML) in Equation 1 was determined on an entry-mean and plot-mean basis as shown in Equations 2, 3, respectively, and are given in Table 1.

(2) $$H^{2}=\frac{\sigma_{H}^{2}}{\sigma_{H}^{2}+\frac{\sigma_{H\,Y}^{2}}{y}+\frac{\sigma_{\varepsilon }^{2}}{y\,r}}$$

(3) $$H^{2}=\frac{\sigma_{H}^{2}}{\sigma_{H}^{2}+\sigma_{H\,Y}^{2}+\sigma_{\varepsilon }^{2}}$$

Where H^2^ represents broad-sense heritability of a given trait. Hybrid, hybrid × year interaction, and error variance components are denoted by $\sigma_{\text{H}}^{2}$, $\sigma_{\text{HY}}^{2}$, and $\sigma_{\varepsilon }^{2}$, respectively. Number of years (*y* = 2) and number of reps per year (*r* = 2) were *y* and *r* in equation 2. Phenotypic correlations were computed using R function *cor* and visualized using corrplot::corrplot. Hierarchical clustering of the EP traits was performed through stats::hclust using Ward’s Minimum Variance (ward.D2). The Ball-Hall Index was used to determine the appropriate number of clusters among the traits. To visualize the dendrogram, the R function *plot* was used with stats::cutree for colorization and ape::as.phylo to convert our object to class *phylo*. Principal component analysis (PCA) was performed in PLINK v1.9 (Purcell et al., 2007) for SNP data and R function *prcomp* for the EP traits. Data visualization was performed with R libraries ggplot2::ggplot, plot3d::text3d, and scatterplot3d::scatterplot3d.

## Results

### Weather Conditions

Weather conditions for 2017 and 2018 as well as the 30-year average from 1988 to 2018 are shown in Supplementary Figure 2. Maximum and minimum air temperature generally follow the 30-year average. Precipitation was found to be more variable across the 30-year average than temperature. In 2017, temperature was characterized by average monthly minimum and maximum typically falling within one standard deviation of the 30-year average. Precipitation was above average throughout much of the growing season. In 2018, average minimum temperatures were below the 30-year average in March and April. Elevated average minimum temperatures began in May. Average monthly maximum temperature was similar to the 30-year average. Precipitation was greater than one standard deviation of the 30-year average for May, June, August, October, and November, and below one standard deviation of the 30-year average for July.

### Validation of Ear Photometry Traits

**FIGURE 1:**
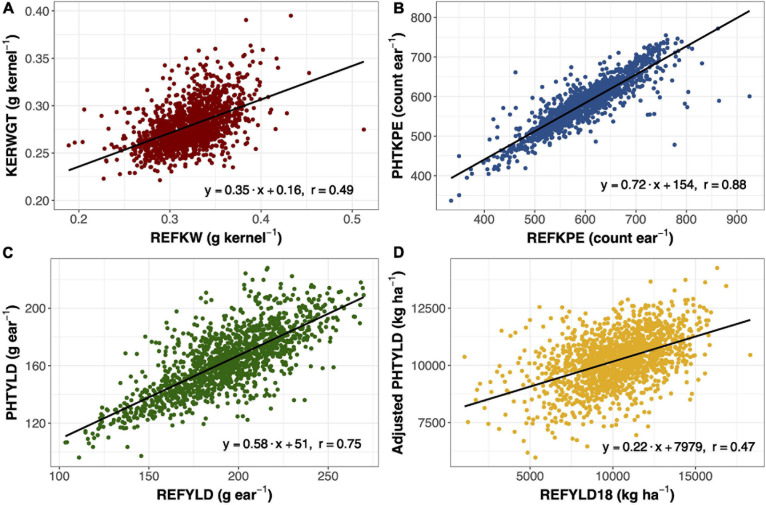
Linear regression and Pearson correlation between reference kernel weight (REFKW) (x-axis) to photometry-estimated kernel weight (KERWGT) (y-axis) in 2017 (*n* = 1,413) **(A)**. Linear regression and Pearson correlation between reference kernels per ear (REFKPE) (x-axis) to photometry-estimated kernels per ear (PHTKPE) (y-axis) in 2017 (*n* = 1,413) **(B)**. Linear regression and Pearson correlation between reference yield (REFYLD) (x-axis) to photometry-estimated yield (PHTYLD) (y-axis) in 2017 (*n* = 1,413) **(C)**. Linear regression and Pearson correlation between reference yield on a plot basis (REFYLD18) and the fitted values for PHTYLD adjusted for stand count in modeling REFYLD18 (REFYLD18 = PHTYLD + Stand Count) in 2018 (*n* = 1,568) **(D)**.

Ear photometry traits that were validated with this dataset include KERWGT, PHTKPE, and PHTYLD. The correlation between KERWGT, PHTKPE, and PHTYLD to their respective reference measurements (REFKW, REFKPE, REFYLD) were *r* = 0.49, *r* = 0.88, and *r* = 0.75, respectively (Figure 1). When the dataset was split based on the background origin, the correlation among KERWGT, PHTKPE, and PHTYLD to their respective reference measurements in temperate germplasm was *r* = 0.51, *r* = 0.89, and *r* = 0.86. In the tropical germplasm these correlations were *r* = 0.38, *r* = 0.89, and *r* = 0.49 (Supplementary Figure 1). PHTYLD was further validated on a per plot basis in 2018. The correlation between PHTYLD and REFYLD18 was *r* = 0.39. Nevertheless, unaccounted variation in the stand count for each plot limited the correlation between these two yield measures. When using stand count as a covariate in the model the correlation between adjusted PHTYLD and REFYLD18 increased to *r* = 0.47 (*r* = 0.54 and *r* = 0.29, in temperate and tropical germplasm, respectively) (Figure 1D).

### Analysis of Ear Photometry Traits

**FIGURE 2:**
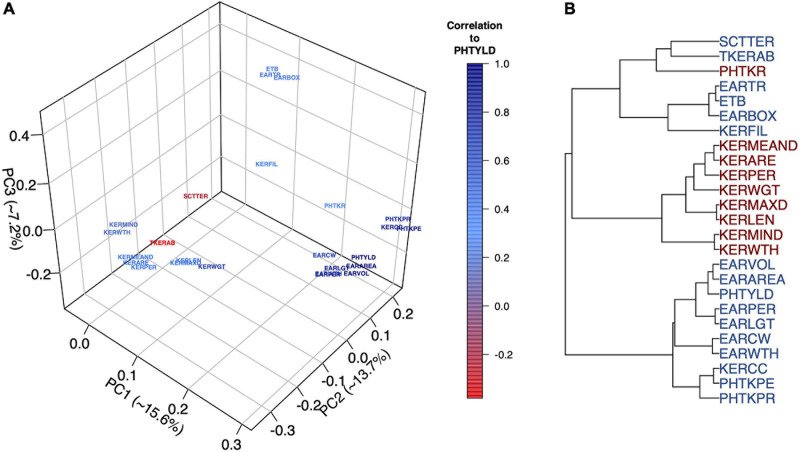
Principal Component Analysis (PCA) to visualize the ear photometry (EP) traits and their correlation to photometry-estimated yield (PHTYLD) **(A)**. Dendrogram displaying the hierarchical clustering of EP traits using Ward’s Minimum Variance. The Ball-Hall index was used to determine the correct number of groups (5) among the EP traits **(B)**.

Multivariate analyses were used to assess the relationship among EP traits including PCA (Figure 2A), hierarchical clustering (Figure 2B), and correlation analysis (Supplementary Figure 3). The Ball-Hall index distinguished five groups in the hierarchical clustering. Traits clustered with PHTYLD were those related to ear size and kernels per ear (EARVOL, EARAREA, EARPER, EARLGT, EARCW, EARWTH, KERCC, PHTKPE, and PHTKPR). Traits regarding the size, shape, and weight of the individual kernels were less correlated with PHTYLD, nevertheless, they were correlated amongst themselves. The remaining clusters relate to the boxiness of the ear, percent of ear filled with kernels, and kernel rows.

PCA was employed on the EP traits to gain a greater understanding of their relationships (Figure 2A). The first three PCs explained 36.5% of the total variance and our interpretations were checked for their orthogonality; beyond PC3, loading factors of the EP traits could not be biologically interpreted. PC1 explained 15.6% of the variation among the traits and was found to separate the traits based on their correlation to yield. PC2 explained 13.7% of the variation and was a contrast between traits involved with increased kernel size and traits that increase kernel number. PC3 explained 7.2% of the variation and was a contrast between the traits that indicated the percent of the ear with kernels and overall ear size.

Broad-sense heritability was estimated for the 25 traits evaluated in this study on an entry-mean and plot-mean basis (Table 1). Among EP traits, entry-mean heritability estimates ranged from 0.42 to 0.75. PHTYLD was among the traits with the lowest entry-mean heritability of 0.52. PHTKPE was marginally more heritable than PHTYLD with a heritability of 0.60. EP traits with an entry-mean heritability greater than or equal to 0.70 include EARLGT, EARPER, KERWTH, KERMEAND, KERMIND, and KERPER. Heritability of the physiology traits measured throughout the growing season and reference yield components ranged from 0.51 to 0.92.

### Population Structure of Germplasm

**FIGURE 3:**
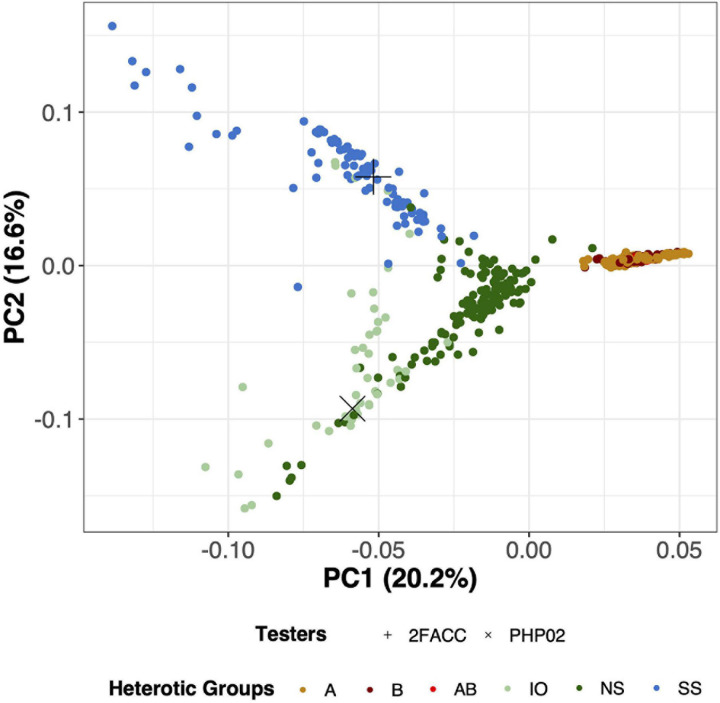
Principal component analysis (PCA) for 533 inbred lines in this study using 755,339 Single Nucleotide Polymorphisms (SNPs). PC1 (x-axis) explains 20.2% of the variation of the SNP data while PC2 (y-axis) explains 16.6% of the variation of the SNP data. Testers in this experiment (2FACC and PHP02) are individually labeled with distinct shapes while heterotic groups are differentiated based on color. A, B, and AB are DTMA heterotic groups, while Iodent (IO), Non-Stiff Stalk (NS), and Stiff Stalk (SS) are temperate heterotic groups.

Through visual assessment using the elbow method, four PCs were found to sufficiently explain the population structure among these inbred lines with 55.5% of the total variation explained in the PCA of SNP data. PC1 explained 20.2% of the total variance and visually separated the temperate and tropical inbred lines (Figure 3). PC2 explained 16.6% of the genomic variation and distinguished the SS from the NS and IO temperate heterotic groups. In PC3, 11.6% of the variance was explained from which the NS and IO heterotic groups could be discriminated (Supplementary Figure 4). 6.5% of the total variance in population structure was explained by PC4 which largely differentiated inbred lines in the NS heterotic group. Within these four PC the heterotic groups of the DTMA inbred lines were never visually separated. When performing PCA on the SNPs from DTMA inbred lines without the temperate material, CIMMYT designated heterotic groups remain difficult to separate (Supplementary Figure 5). Thus, our analysis does not separate the DTMA inbred lines into known heterotic groups.

### Analysis of Heterotic Patterns Through Ear Photometry

The heterotic patterns of the temperate and tropical inbred lines were evaluated for their testcross performance with 2FACC (Table 2) and PHP02 (Table 3). In these tables, the heterotic groups were presented as the mean and range for each trait. Considerable differences were found within and among the heterotic groups based on the means and ranges of the phenotypic traits.

**TABLE 2:** Best linear unbiased predictions (BLUPs) of all traits measured in this study for inbred lines crossed to 2FACC based on heterotic group, NS (Non-Stiff Stalk), SS (Stiff Stalk), and IO (Iodent).

**Trait Sections**	**Trait**	**Units**	**Significance**	**NS (*n* = 146)**		**IO (*n* = 48)**		**SS (*n* = 104)**	
Reference Physiology	AD	GDD*	***	709.9 (656.1–760.1)	b	699.3 (676.1–750.4)	c	719.9 (666.3–757.9)	a
	SD	GDD	***	720.3 (659.2–774.7)	b	711.7 (689.2–763.9)	c	732.5 (673–779.5)	a
	ASI	GDD	*	68.7 (52.3–93.9)	b	70.3 (58.7–93.7)	ab	71.5 (53.3–107.3)	a
	ASI	Days	ns	1.2 (−0.4–2.3)		1.3 (0.8–2.3)		1.3 (0–2.9)	
	PH	Cm	**	223.9 (189.7–250)	a	217.8 (193.5–235.3)	b	219.9 (187.3–244.8)	b
	EH	Cm	ns	93.7 (71.6–124.4)		93.6 (77.4–109.1)		95 (71.4–122.1)	
Reference Yield Components	REFKW	g kernel^–1^	*	0.324 (0.262–0.45)	a	0.318 (0.277–0.342)	b	0.318 (0.252–0.356)	b
	REFKPE	count ear^–1^	***	626.7 (422.7–792.6)	a	596 (476.7–706.9)	b	562.8 (396.6–735.1)	c
	REFYLD	g ear^–1^	***	201.5 (151.2–234.8)	a	188.9 (153.5–227.7)	b	179 (138.1–209.1)	c
	REFYLD18‡	kg ha^–1^	***	10,334.8 (7,155–12,484.8)	a	9,567.8 (7,718.4–11,224.3)	b	9,556 (6,727.2–14,118.8)	b
	MOISTURE‡	%	*	15 (14.5–16.4)	a	14.9 (14.5–16.5)	b	15 (14.6–16.4)	ab
Ear Photometry	PHTYLD	g ear^–1^	***	162.2 (141.3–193.7)	a	156.4 (142.7–175)	b	151.7 (129–173.9)	c
	PHTKPE	count ear^–1^	***	597 (480.4–681.7)	a	575.1 (524.5–635.6)	b	556.5 (438.9–648)	c
	KERWGT	g kernel^–1^	ns	0.272 (0.252–0.315)		0.271 (0.254–0.287)		0.27 (0.249–0.291)	
	EARAREA	cm^2^ ear^–1^	***	88.8 (76.5–100.9)	a	85.4 (77–96.7)	b	82.4 (69.2–92.7)	c
	EARBOX	–	***	0.846 (0.829–0.864)	a	0.843 (0.819–0.855)	a	0.84 (0.817–0.857)	b
	EARCW	–	***	5.12 (4.61–5.76)	a	4.96 (4.77–5.19)	b	5.02 (4.67–5.37)	b
	EARLGT	cm ear^–1^	***	19.6 (17.3–22.8)	a	19.4 (17.3–21.8)	a	18.5 (15.5–20.3)	b
	EARPER	cm ear^–1^	***	49.7 (44.8–55.7)	a	48.9 (44.1–54.6)	a	46.8 (40.2–51.2)	b
	EARTR	cm^2^ ear^–1^	***	81.9 (77.4–85.7)	a	81.1 (75.8–83.8)	b	81.1 (75.5–84.6)	b
	EARVOL	cm^2^ ear^–1^	***	373.3 (313–435.7)	a	352.4 (322.6–406.6)	b	343.9 (292.9–394.2)	c
	EARWTH	cm ear^–1^	***	5.34 (4.96–5.9)	a	5.2 (5–5.43)	b	5.25 (4.91–5.58)	b
	ETB	–	***	69.3 (64.1–74.2)	a	68.4 (62.1–71.6)	b	68.1 (61.6–72.5)	b
	KERARE	cm^2^ kernel^–1^	ns	0.337 (0.305–0.427)		0.339 (0.311–0.362)		0.339 (0.306–0.377)	
	KERCC	–	***	153 (121.5–176)	a	146.5 (134.7–162.9)	b	143.9 (111.2–163.3)	b
	KERFIL	cm^2^ (cm^2^)^–1^	***	86.5 (78.3–89.2)	b	86.9 (84.7–89.9)	b	87.6 (83.2–90.4)	a
	KERLEN	cm kernel^–1^	ns	0.867 (0.793–0.939)		0.866 (0.81–0.908)		0.861 (0.81–0.908)	
	KERMAXD	cm kernel^–1^	ns	0.893 (0.822–0.962)		0.892 (0.837–0.933)		0.887 (0.837–0.933)	
	KERMEAND	cm kernel^–1^	ns	0.639 (0.603–0.734)		0.641 (0.615–0.662)		0.641 (0.608–0.68)	
	KERMIND	cm kernel^–1^	*	0.435 (0.394–0.572)	b	0.438 (0.419–0.462)	ab	0.441 (0.404–0.483)	a
	KERPER	cm kernel^–1^	ns	2.41 (2.24–2.73)		2.4 (2.25–2.5)		2.4 (2.25–2.55)	
	KERWTH	cm kernel^–1^	*	0.454 (0.416–0.605)	b	0.455 (0.438–0.478)	ab	0.459 (0.423–0.506)	a
	PHTKR	count ear^–1^	***	17.3 (15.5–19.6)	a	16.9 (15.5–18.5)	b	17.1 (15.8–18.4)	ab
	PHTKPR	count ear^–1^	***	40.4 (32.4–47)	a	39.5 (34.9–45.4)	b	37.6 (29.1–42.3)	c
	SCTTER	cm^2^ (cm^2^)^–1^	***	11.5 (8.7–18.6)	a	10.8 (8–13.1)	b	10.3 (7.7–15)	b
	TKERAB	cm cm^–1^	**	6.5 (4.5–9.6)	b	7.1 (5–10.4)	a	6.7 (4.9–10.3)	ab

Given in terms of mean for the heterotic group with the range in parenthesis. Letters following the BLUPs indicate significant differences between heterotic groups at *p* value < 0.05. The same letters signify no significant differences between groups. *Daily Growing Degree Days (C°) = $\frac{T_{\text{max}}\,C-T_{\text{min}}\,C}{2}-10\,\text{C}$ If Max Temp > 30°C, then Max Temp = 30°C. If Max Temp < 10°C, then Max Temp = 10°C. If Min Temp > 30°C, then Min Temp = 30°C. If Min Temp < 10°C, then Min Temp = 10°C. ^†^Only measured in 2017. For these traits, the effect of Year was removed from the model. ^‡^Only measured in 2018. For these traits, the effect of Year was removed from the model.

**TABLE 3:** Best linear unbiased predictions (BLUPs) of all traits measured in this study for inbreds crossed to PHP02 based on heterotic group, NS (Non-Stiff Stalk), SS (Stiff Stalk), IO (Iodent), and DTMA (Drought Tolerant Maize for Africa).

**Trait Sections**	**Trait**	**Units**	**Significance**	**NS (*n* = 151)**		**IO (*n* = 46)**		**SS (*n* = 89)**		**DTMA (*n* = 247)**	
Reference Physiology	AD	GDD*	***	704.4 (704.4–761.3)	b	702.4 (670.1–747.9)	b	696.7 (652.9–751.7)	b	774.7 (699.6–856.9)	a
	SD	GDD	***	720.8 (720.6–770.7)	b	727.5 (678.1–846.8)	b	714.5 (676.1–754.3)	b	804.9 (713.4–956)	a
	ASI	GDD	***	74.4 (72.5–107.5)	c	80 (62.2–111.2)	b	76.5 (56.9–104.5)	bc	85.5 (58.2–124.4)	a
	ASI	Days	***	1.5 (1.4–3.2)	c	1.9 (0.9–9.7)	b	1.5 (0.6–2.9)	bc	2.3 (0.7–6.9)	a
	PH†	Cm	***	221.1 (221.3–258.3)	b	205.9 (171.6–250.3)	c	223.5 (183.5–255.5)	b	254.9 (216.5–312.3)	a
	EH†	Cm	***	101.6 (101.7–130.7)	b	93.9 (66.1–120.2)	c	103.1 (79–125.3)	b	133.3 (100.8–175.1)	a
Reference Yield Components	REFKW†	g kernel^–1^	***	0.334 (0.247–0.383)	a	0.318 (0.268–0.364)	b	0.335 (0.297–0.377)	a	0.338 (0.273–0.41)	a
	REFKPE†	count ear^–1^	***	618.9 (472.4–799.4)	a	562.4 (451.5–714.8)	c	620.9 (503.3–737.4)	a	597.5 (399.1–730.9)	b
	REFYLD†	g ear^–1^	***	206.6 (133.8–252.3)	a	180.5 (132.8–241.5)	b	207.3 (153–257.3)	a	201.4 (141.2–240.7)	a
	REFYLD18‡	kg ha^–1^	***	10,054.1 (4828.2–16,128.4)	b	8,885.1 (5,717.2–13,035.7)	c	10,529.2 (8,342.4–13,726.5)	a	8,598.6 (5,174.6–12,161.3)	c
	MOISTURE‡	%	***	14.9 (10.2–16)	b	14.8 (14.2–15.4)	b	14.9 (10.3–16.1)	b	16.6 (14.9–20.5)	a
Ear Photometry	PHTYLD	g ear^–1^	***	167.1 (132.9–192)	a	154 (131.1–195.2)	b	166.7 (140.3–185.7)	a	165.6 (128.7–194)	a
	PHTKPE	count ear^–1^	***	601.9 (505.6–688.9)	a	562.2 (473–718.7)	c	604.8 (530.6–686.5)	a	582.8 (423.5–682.3)	b
	KERWGT	g kernel^–1^	***	0.278 (0.251–0.312)	b	0.272 (0.256–0.287)	c	0.277 (0.26–0.299)	b	0.284 (0.255–0.327)	a
	EARAREA	cm^2^ ear^–1^	***	91.4 (72–104.7)	a	83.7 (70.5–102)	b	91.5 (76.5–99.6)	a	92 (71.3–104.7)	a
	EARBOX	–	***	0.854 (0.832–0.871)	a	0.85 (0.83–0.864)	ab	0.85 (0.838–0.866)	b	0.845 (0.803–0.869)	c
	EARCW	–	***	5 (4.56–5.56)	a	4.84 (4.53–5.25)	b	5.04 (4.65–5.41)	a	5.02 (4.6–5.41)	a
	EARLGT	cm ear^–1^	***	20.4 (17–23.8)	a	19.2 (16.8–22.2)	b	20.4 (17.8–22.3)	a	20.6 (16.9–25)	a
	EARPER	cm ear^–1^	***	51.5 (43.1–57.9)	b	48.3 (42.7–55.7)	c	51.3 (45.1–56.6)	b	52.5 (44.6–67.6)	a
	EARTR	cm^2^ ear^–1^	***	83.9 (80.3–86.7)	a	83.4 (78.8–85.9)	ab	82.8 (80.2–86)	b	82.1 (75.9–86.7)	c
	EARVOL	cm^2^ ear^–1^	***	378 (284.4–453.1)	a	341 (278.8–435.4)	b	380.6 (304.6–424.3)	a	383.3 (296.8–451.4)	a
	EARWTH	cm ear^–1^	***	5.24 (4.75–5.81)	a	5.08 (4.75–5.52)	b	5.29 (4.88–5.57)	a	5.28 (4.85–5.67)	a
	ETB	–	***	71.7 (67.1–75.6)	a	71.1 (65.3–74.3)	ab	70.5 (67.3–74.6)	b	69.4 (61.7–75.6)	c
	KERARE	cm^2^ kernel^–1^	***	0.352 (0.315–0.398)	b	0.346 (0.318–0.369)	b	0.349 (0.316–0.391)	b	0.365 (0.307–0.443)	a
	KERCC	–	***	155.3 (125.5–185.1)	a	144.6 (119.5–184.5)	c	157.1 (136.7–178.5)	a	152.2 (111.5–179.6)	b
	KERFIL	cm^2^ (cm^2^) ^–1^	***	86.5 (75.8–89.3)	a	87.3 (83.3–89.9)	a	86.5 (79.5–88.8)	a	85.1 (76.5–90.6)	b
	KERLEN	cm kernel^–1^	***	0.872 (0.8–0.949)	b	0.854 (0.794–0.909)	c	0.866 (0.832–0.934)	b	0.882 (0.817–0.966)	a
	KERMAXD	cm kernel^–1^	***	0.898 (0.83–0.974)	b	0.881 (0.823–0.934)	c	0.893 (0.857–0.96)	bc	0.91 (0.849–0.989)	a
	KERMEAND	cm kernel^–1^	***	0.655 (0.619–0.703)	b	0.651 (0.624–0.675)	b	0.653 (0.619–0.696)	b	0.67 (0.61–0.746)	a
	KERMIND	cm kernel^–1^	***	0.454 (0.408–0.507)	b	0.458 (0.426–0.487)	b	0.453 (0.42–0.51)	b	0.47 (0.41–0.56)	a
	KERPER	cm kernel^–1^	***	2.44 (2.28–2.72)	b	2.4 (2.26–2.5)	c	2.42 (2.29–2.58)	bc	2.54 (2.36–2.97)	a
	KERWTH	cm kernel^–1^	***	0.471 (0.424–0.524)	b	0.473 (0.442–0.507)	b	0.468 (0.434–0.523)	b	0.49 (0.429–0.592)	a
	PHTKR	count ear^–1^	***	16.9 (15.3–19)	b	16.6 (15.4–18.3)	b	17.2 (15.6–18.4)	a	16.8 (15.2–19.6)	b
	PHTKPR	count ear^–1^	***	41.3 (34–48.2)	a	38.5 (32.4–45.8)	c	40.9 (37–45.1)	a	39.9 (28.2–45)	b
	SCTTER	cm^2^ (cm^2^) ^–1^	***	11.8 (9.1–22.9)	b	10.9 (8.3–15.2)	b	11.7 (9.3–19)	b	12.9 (7.4–21.7)	a
	TKERAB	cm cm^–1^	***	5.2 (3.5–8.2)	c	5.5 (3.8–7.4)	bc	5.9 (3.5–8)	ab	6.1 (3.2–10.8)	a

Given in terms of mean for the heterotic group with the range in parenthesis. Letters following the BLUPs indicate significant differences between heterotic groups at *p* value < 0.05. The same letters signify no significant differences between groups. *Daily Growing Degree Days (C°) = $\frac{T_{\text{max}}\,C-T_{\text{min}}\,C}{2}-10\,\text{C}$ If Max Temp > 30°C, then Max Temp = 30°C. If Max Temp < 10°C, then Max Temp = 10°C. If Min Temp > 30°C, then Min Temp = 30°C. If Min Temp < 10°C, then Min Temp = 10°C. ^†^Only measured in 2017. For these traits, the effect of Year was removed from the model. ^‡^Only measured in 2018. For these traits, the effect of Year was removed from the model.

**FIGURE 4:**
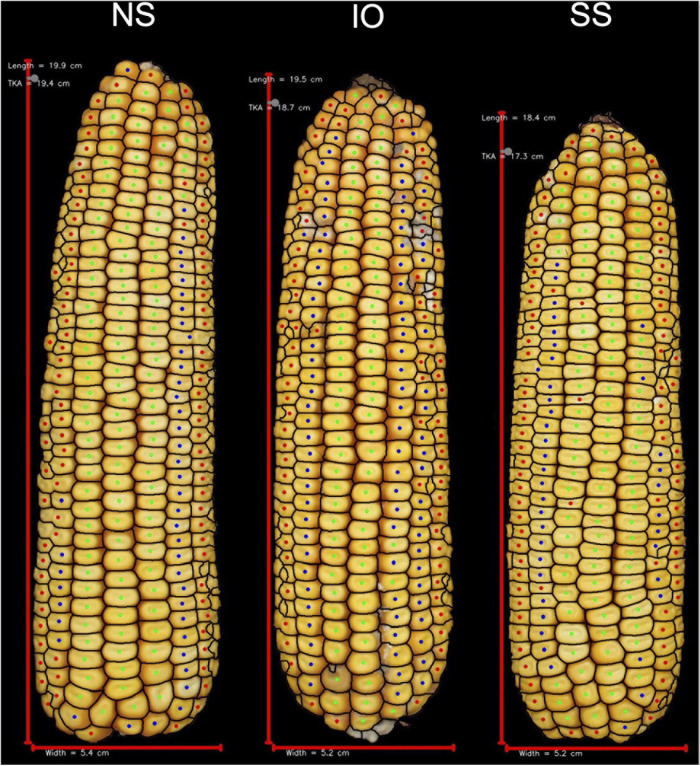
Representative ears for each of the heterotic groups in hybrid combination with 2FACC. Selected ears had an average yield and yield components for their heterotic group combination. Abbreviations for heterotic groups include: NS (Non-Stiff Stalk), IO (Iodent), and SS (Stiff Stalk).

A decrease in testcross performance was seen when 2FACC was crossed within the SS heterotic group compared to NS and IO (Table 2 and Figure 4). Traits significantly (*p* value < 0.05) reduced included PHTYLD, PHTKPE, EARAREA, EARBOX, EARLGT, EARPER, EARVOL, PHTKPR, REFKPE, and REFYLD. There was also a significant (*p* value < 0.05) reduction in heterotic potential of the IO heterotic group as compared to NS in testcrosses to 2FACC for PHTYLD, PHTKPE, EARAREA, EARCW, EARVOL, EARWTH, ETB, KERCC, PHTKR, PHTKPR, SCTTER, REFKW, REFKPE, REFYLD, REFYLD18, MOISTURE, AD, SD, and PH. Traits KERWTH, KERMIND, AD, SD, and ASI (GDD) were the only traits significantly greater in the SS as compared to NS heterotic group with IO material as an intermediate not significantly different from either SS or NS for KERMIND, KERWTH, and ASI. In testcross performance with 2FACC, kernel attributes were generally not significantly improved as a result of heterosis.

Many agronomic and EP traits were significantly reduced when PHP02 was crossed within the IO heterotic group as compared to SS and NS heterotic groups. Traits significantly (*p* value < 0.05) reduced were PHTYLD, PHTKPE, KERWGT, EARAREA, EARCW, EARLGT, EARPER, EARVOL, EARWTH, KERCC, KERLEN, PHTKPR, PH, and EH (Table 3). SS and NS heterotic groups were only significantly (*p* value < 0.05) different for EARBOX, EARTR, ETB, PHTKR, TKERAB, and REFYLD18 when crossed to PHP02.

**FIGURE 5:**
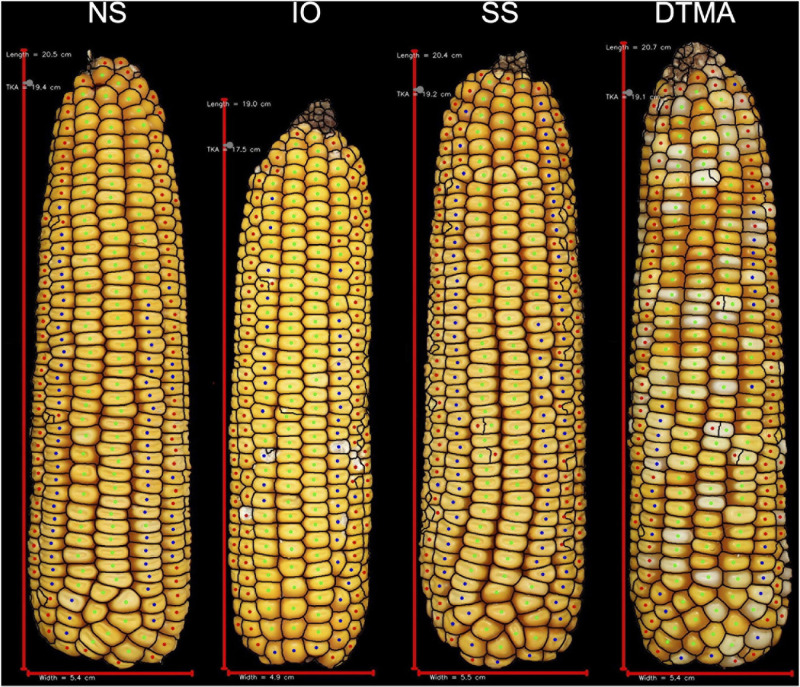
Representative ears for each of the heterotic groups in hybrid combination with PHP02. Selected ears had an average yield and yield components for their heterotic group combination. Abbreviations for heterotic groups include: NS (Non-Stiff Stalk), IO (Iodent), SS (Stiff Stalk), and DTMA (Drought Tolerant Maize for Africa).

In testcross performance with PHP02, the DTMA inbred lines exhibited similar performance to SS and NS heterotic backgrounds (Figure 5). PHTYLD, EARAREA, EARCW, EARLGT, EARVOL, EARWTH, REFKW, and REFYLD were not significantly different from the SS and NS heterotic groups; however, PHTKPE, EARBOX, EARTR, ETB, KERCC, KERFIL, PHTKPR, REFKPE, and REFYLD18 were significantly (*p* value < 0.05) reduced. The reduction in kernel number attributes was present due to a significant (*p* value < 0.05) increase in SCTTER compared to NS and SS heterotic groups and TKERAB compared to NS. PHTYLD was not significantly different between SS, NS, and DTMA heterotic groups though due to a significant (*p* value < 0.05) increase in KERWGT, EARPER, KERARE, KERLEN, KERMAXD, KERMEAND, KERMIND, KERPER, and KERWTH in DTMA compared to NS and SS inbred lines. In addition to EP traits, hybrids from DTMA inbred lines exhibited significantly (*p* value < 0.05) greater AD, SD, ASI (GDD and Days), PH, and EH as expected due to their tropical origin.

Variance of PHTYLD was estimated on a per plot basis from 10 ears in 2017 and five ears in 2018. Due to the differences in number of ears sampled per plot, analysis was not combined between years. In 2017, mean within-plot variance per plot was 427.5. DTMA testcrosses had significantly greater (*p* value < 0.001) within-plot variance than plots of temperate descent (510.2 and 392, respectively). In 2018, mean within-plot variance per plot was 316.3. DTMA testcrosses had significantly greater (*p* value < 0.05) within-plot variance than temperate testcrosses (351.6 and 302.5, respectively).

## Discussion

### Ear Photometry

Grain yield as measured on a per plot basis has been the primary trait selected upon in commercial breeding programs. As a complex trait, grain yield is a composite of many yield-related traits known as yield components. While yield components generally are found to be more heritable (Table 1), phenotyping these traits has historically been time-consuming, labor-intensive, prone to error, and difficult to scale in a large breeding program (Bernardo, 2014; Cooper et al., 2014). Ear photometry removes many of these barriers while providing a more in-depth understanding of yield and yield components.

Photometry-estimated yield was validated in this study with regards to reference yield measured from a combine in 2018 (*n* = 1,568) and on a per ear basis in 2017 (*n* = 1,413). The correlation between photometry-estimated yield and reference yield in 2018 was *r* = 0.39. When stand count was used as a covariate in the model, the correlation increased to *r* = 0.47 (Figure 1D). On a per ear basis, yield is the product of kernel number and average kernel size. The correlation between photometry-estimated yield per ear and reference yield per ear was *r* = 0.75 (Figure 1C).

Yield components, kernels per ear and kernel weight, were other traits that were validated in 2017 (*n* = 1413) (Figure 1). Kernel number per ear was taken at the plot level and divided by 10, the number of ears taken per plot. Kernel weight was determined by dividing the total kernel weight by the total kernel number. Photometry-estimated kernels per ear was correlated with reference kernels per ear (*r* = 0.88) (Figure 1B). Photometry-estimated kernel weight was correlated with reference kernel weight (*r* = 0.49) (Figure 1A). The correlation between photometry-estimated kernels per ear and reference kernels per ear was unaffected by the background of the germplasm, while the correlation between photometry-estimated kernel weight and reference kernel weight was reduced in the tropical (*r* = 0.38) compared to the temperate germplasm (*r* = 0.51) (Supplementary Figure 1). As such, the correlation of photometry-estimated yield to reference yield fell in the tropical germplasm (*r* = 0.49) as compared to the temperate germplasm (*r* = 0.86). Pioneer Hi-Bred International also validated many of the traits in this platform including kernels per ear (*R*^2^ = 0.87; *n* = 287) and yield (*R*^2^ = 0.97; *n* = 1,500) in temperate germplasm (Hausmann et al., 2009). This study shows that EP can be extended to quantify variation in ear traits in tropical germplasm.

Grift et al. (2017), Miller et al. (2017), and Makanza et al. (2018) previously evaluated high-throughput methods for yield component assessment in maize. Grift et al. (2017) used machine learning to evaluate kernel number per year on 23 maize ears. Using the full ear, they found errors ranging from −7.67 to 8.60% which indicated under- and over-counting, respectively, and a coefficient of determination of *R*^2^ = 0.7. Miller et al. (2017) evaluated the yield components of 445 diverse inbred lines. In their platform, three types of images were obtained. First, each genotype had three ears imaged from two angles where the second angle was a 90-degree rotation of the ear. After being shelled, these cobs were again imaged. Finally, the kernels were imaged when spread out on a black sheet. Using this platform, these authors found high correlations between ear length and kernel length to their reference phenotypes with coefficients of determination of *R*^2^ = 0.99 and 0.74, respectively. Makanza et al. (2018) evaluated 10 hybrids from an experiment performed in Zimbabwe. Ears were collected from these field trials, arranged on a black cloth, and were photographed from a mounted camera tripod stand. Shelled kernels were imaged on a black background. Yield components related to the ears (i.e., ear length and ear width) were accurately correlated to reference measurements (*r* = 0.99 and 0.97, respectively). Yield components such as kernel count and kernel weight were also correlated to their reference measurements (*r* = 0.99 and 0.94, respectively).

The phenotyping platform described in this paper presents a potential improvement over the previously mentioned phenotyping strategies as the kernels do not need to be shelled from the ears and the ears are only imaged from one angle. The ease of using this platform enables it to be scaled to the level needed in a breeding program; however, the accuracy of kernel weight assessment was reduced in this study from the platform described in Makanza et al. (2018) as the kernels were not shelled prior to imaging.

Heritability of a given trait is a function of the germplasm under evaluation and the effect of the environment (Bernardo, 2014). In our study, heritability of photometry-estimated yield was among the lowest traits evaluated (*H*^2^ = 0.52). Lian et al. (2014) evaluated the heritability of multiple traits in 969 maize biparental crosses. They found that heritability of grain yield ranged from 0.17 to 0.92 with a mean of 0.46. In our study, the heritability of many yield components was increased in comparison to photometry-estimated yield (Table 1). Photometry-estimated traits such as kernels per ear (*H*^2^ = 0.60), ear length (*H*^2^ = 0.71), and kernel perimeter (*H*^2^ = 0.75) are a subset of yield components with an increased heritability. Ross et al. (2006) evaluated heritability of yield components and found that ear length and kernel row number were more heritable than grain yield. In their study which used a biparental population, kernel attributes such as kernel length, kernel width, kernel thickness, and 100-kernel weight had heritabilities ranging from 0.65 to 0.79. Selection based on these traits with increased heritability could improve genetic gain and selection accuracy (Araus et al., 2018).

Variation between plots is the foundation of plant breeding, indicating sources of genetic variation from which breeders make selections. Variation within plots is measured relatively less often as grain yield is often estimated at a plot level. Nevertheless, within-plot variability has been suggested to be an indicator for yield stability in varying environments (Hausmann et al., 2009). In both of these years, testcrosses to DTMA inbred lines resulted in a significant (*p* value < 0.001, 2017; *p*-value *<* 0.05, 2018) increase in within-plot variance compared to temperate testcrosses. Many of the temperate inbred lines used in this study were the result of intensive selection where inbred lines were evaluated in multi-environment trials within commercial breeding programs where yield stability was an important consideration (Cooper et al., 2014). Additionally, the unadapted nature of the DTMA inbred lines to the United States Corn Belt could have been an extra source of the within-plot variation. However, the range in variability in the DTMA material was 23–2,866 in 2017 and 12–3,166 in 2018 suggesting variability in the yield stability of these testcrosses.

### Description of Heterotic Groups

Principal component analysis is commonly employed to assess population structure in genomic studies (Figure 3). The heterotic groups of the temperate inbred lines were classified in accordance with Beckett et al. (2017). From canonical axes 2 and 3 (16.6 and 11.6% of the total variation explained, respectively) of the principal component analysis, Stiff Stalk, Non-Stiff Stalk, and Iodent heterotic groups were visually separated (Supplementary Figure 4). The Stiff Stalk heterotic group was first separated from the Non-Stiff Stalk and Iodent inbred lines suggesting that the Non-Stiff Stalk and Iodent heterotic groups are more closely related than inbred lines of Stiff Stalk origin as previously reported (Mikel, 2008; Nelson et al., 2008; Beckett et al., 2017).

Mikel (2008) evaluated the genetic diversity of 55 inbred parents used in Holden’s Foundation Seeds and Pioneer Hi-Bred International. They classified two major heterotic groups among temperate germplasm: Stiff Stalk and Non-Stiff Stalk. Through pedigree-based records, Mikel and Dudley (2006) and Mikel (2008) trace the lineage of the Stiff Stalk heterotic group to public inbred line B73 and conclude there is less genetic diversity within Stiff Stalk material than Non-Stiff Stalk material. In evaluating the Non-Stiff Stalk material, subgroups included germplasm derivatives from Lancaster Sure Crop, Minnesota 13, Leaming Corn, Northwestern Dent, and Iodent (Troyer, 1999; Mikel, 2008). The role of Iodent germplasm has increased in commercial programs and hybrids composed of Iodent and Non-Stiff Stalk inbred parents are commercially viable (Mikel, 2011) leading to its own designation in this study as has previously been done (Nelson et al., 2008; Beckett et al., 2017; White et al., 2020).

The temperate and tropical inbred lines could be visually separated along principal component 1 (20.2%) (Figure 3). Multiple heterotic groups are represented within the CIMMYT breeding program (personal communication), but their classification was difficult to distinguish in PCA with (Figure 3) and without the temperate material (Supplementary Figure 5) as has previously been reported by Wu et al. (2016). Additionally, inclusion into United States breeding programs did not appear to be dependent on their tropical heterotic group classification. Based on the variation in the genotypic information, we believe that the DTMA germplasm could have potential in hybrid combination with all temperate heterotic groups. Holland and Goodman (1995) report similar findings of broad utility of several exotic families to temperate heterotic groups.

### Phenotypic Characteristics of Heterotic Groups

Since maize in the United States is commercially grown as a hybrid crop, inbred lines are normally selected based on testcross rather than *per se* performance (Bernardo, 2014). In this study, inbred testers PHP02 (Iodent) and 2FACC (Stiff Stalk) were used to characterize the heterotic patterns and ear phenotypes of available inbred lines. The implications of heterosis were evident when inter- and intra-heterotic group crosses were compared (Tables 2, 3 and Figures 4, 5). Within the crosses to PHP02, Iodent inbred lines were significantly reduced as compared to Stiff Stalk and Non-Stiff Stalk material with regards to traits pertaining to yield, ear size, kernels per ear, and kernel size (Table 3). Within crosses to 2FACC, Stiff Stalk inbred lines were significantly reduced for yield, ear size, and kernels per ear; however, many kernel size traits were not significantly reduced (Table 2). The effects of heterosis were more noticeable in traits related to yield, ear size, and kernel number than kernel size. Stiff Stalk and Non-Stiff Stalk groups were found to have similar heterotic potential when crossed with Iodent tester PHP02 (Table 3 and Figure 5), while Stiff Stalk tester 2FACC was found to combine best with Non-Stiff Stalk inbred lines with the Iodent heterotic group being an intermittent improvement to the Stiff Stalk heterotic group (Table 2 and Figure 4). As Plant Variety Protection expires on inbred lines, ear photometry can provide valuable information about these inbred lines which are considered as sources of new germplasm in breeding programs without previous access to the proprietary material.

**FIGURE 6:**
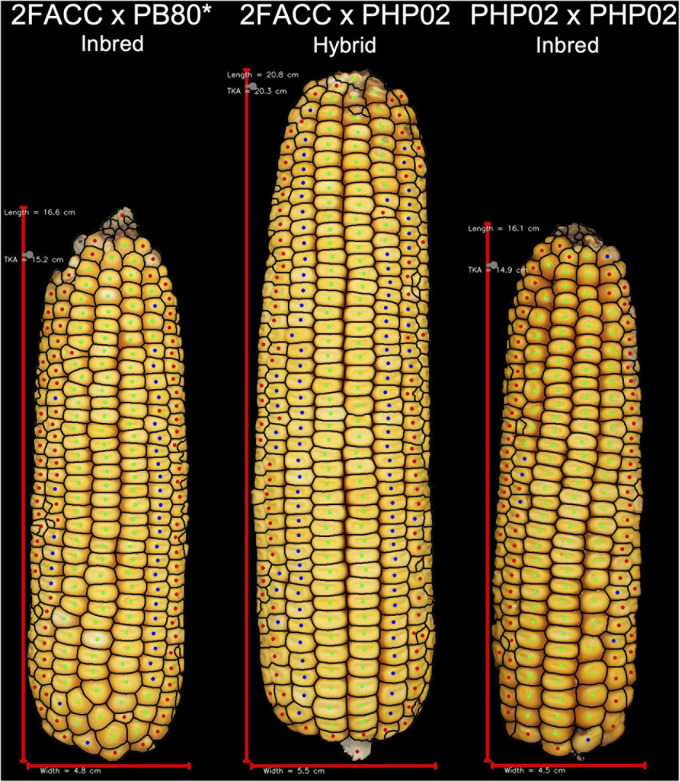
Visual description of hybrid breeding. Inbred line 2FACC, on the left, from the Stiff Stalk heterotic group and inbred line PHP02, on the right, from the Iodent heterotic group show reduced vigor due to inbreeding depression. The hybrid of these inbred lines displays greater yield potential than either inbred parent due to hybrid vigor. *2FACC inbred was not produced in this study. As the progenitor of 2FACC, PB80 produced a highly inbred line when crossed to 2FACC.

Heterosis and hybrid vigor are the foundations for the success of modern maize breeding in the United States (Figure 6). Hauck et al. (2014) found that the effects of heterosis were apparent in many yield components including kernel row number, kernel weight, and kernels per row. Additionally, in evaluating midparent heterosis, Tollenaar et al. (2004) found the heterotic effect of kernels per area to be greater than kernel weight. While primarily measured at harvest, these yield components are determined throughout the growing season. Maximum kernel number per ear is determined in the vegetative growth stages with optimum growth conditions maximizing this yield component. Subsequently, kernel weight is a function of the number of kernels on a given ear and the amount of resources that are allocated to the reproductive organs in their critical period of grain filling following pollination (Nielsen, 2002). Average kernel weight is the more elastic yield component in comparison with kernel number per ear which leads to its heritability (Table 1) and midparent heterosis (Tollenaar et al., 2004) being reduced. The effect of heterosis and genetic gain in physiological processes of maize development is the foundation for greater grain yield (Tollenaar and Lee, 2006).

In hybrid combination with PHP02, the tropical germplasm performed well for many ear traits including yield, kernels per ear, and ear length in this study. Tropical germplasm was previously considered for inclusion into commercial germplasm pools (Holland and Goodman, 1995; Mikel, 2011). Holland and Goodman (1995) found that several semiexotic topcrosses were comparable in yields compared to B73Ht × Mo17Ht F_1_ hybrids. These results indicate the potential of these lines to simultaneous increase genetic diversity and grain yield upon intensive plant breeding efforts. Exotic germplasm was previously used in commercial breeding programs. For example, inbred PHG39, a main contributor of the contemporary Pioneer Hi-bred International Stiff Stalk heterotic group, is comprized of 25% exotic germplasm (Maize Amargo) (Mikel and Dudley, 2006; Mikel, 2011). Pre-breeding efforts are needed on traits such as plant height, ear height, and growing degree days to flowering to adapt these inbred lines to production in the United States Corn Belt.

## Conclusion

Ear photometry methods can be used to identify and quantify traits that were previously difficult to measure at scale in a breeding program. In this study, kernels per ear (*r* = 0.88) and kernel weight (*r* = 0.49) were both correlated with their reference measurements. Grain yield on per ear basis and plot basis were also correlated with reference measurements with correlations of *r* = 0.75 and *r* = 0.47, respectively. Twenty-five ear traits were assessed. Traits related to ear size and kernels per ear were found to be more related to yield than kernel attributes. Similarly, traits related to ear size and kernels per ear were found to be affected by heterosis to a greater degree than kernel size when evaluating inter-heterotic group crosses compared to intra-heterotic group crosses. Yield components were generally found to be more heritable than grain yield indicating their potential in inbred selection. Temperate, commercial United States heterotic groups had a wide range of phenotypes when inter- and intra-heterotic group testcrosses were evaluated. DTMA inbred lines, when evaluated using an Iodent tester, were found to have comparable yields to temperate material due to an increase in kernel weight that overcame the decrease in kernels per ear. Detailed phenotypic description of inbred lines is instrumental in the use of ex-PVP inbreds in public breeding programs and the incorporation of diverse germplasm to sustain long-term genetic gain in the commercial United States maize industry.

## Data Availability Statement

The datasets presented in this study can be found in online repositories. The names of the repository/repositories and accession number(s) can be found below: Best linear unbiased predictions (BLUPs) for the 25 yield-related traits measured by ear photometry for the 831 testcross hybrids evaluated in this study can be found in the Purdue University Research Repository, https://purr.purdue.edu/publications/3623/1.

## Author Contributions

MT initiated, conceived, and coordinated all the experiments. ST and AS performed most of the experiments. ST wrote the manuscript with contributions of all the authors. All authors contributed to the article and approved the submitted version.

## Conflict of Interest

AS was employed by the company Advanta Seeds. The remaining authors declare that the research was conducted in the absence of any commercial or financial relationships that could be construed as a potential conflict of interest.

## Footnotes

1 http://hdl.handle.net/11529/10548156
