# Supplementary material for: Heterotic Patterns of Temperate and Tropical Maize by Ear Photometry
Source: Front Plant Sci. 2021 Jun 14;12:616975. doi: 10.3389/fpls.2021.616975 (PMC8238002; doi:10.3389/fpls.2021.616975)
Supplement: Supplementary file 1 [file Data_Sheet_1.docx]

**
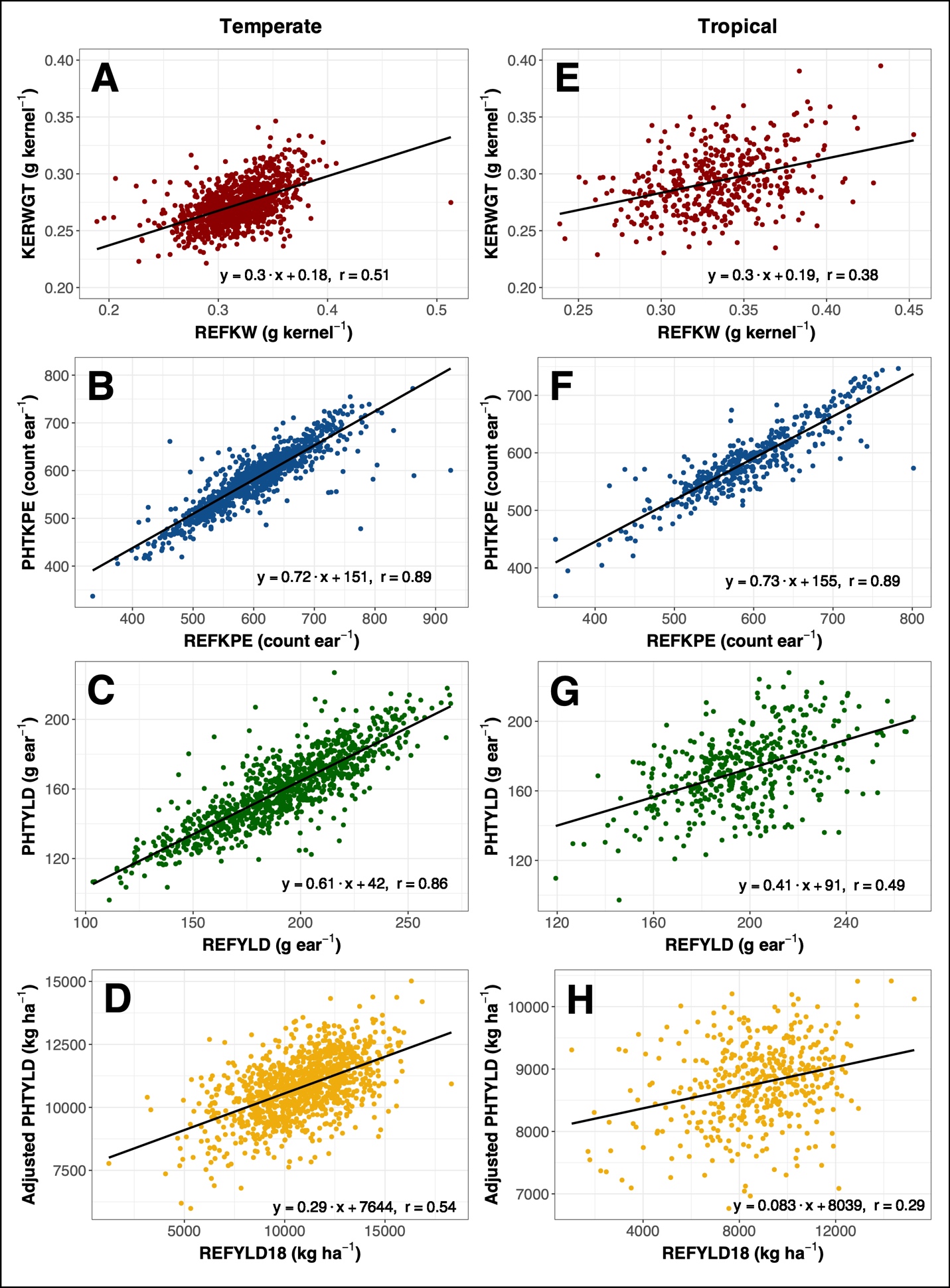
**

**Supplemental Figure 1.** Linear regression and Pearson correlation for kernel weight, kernels per ear, grain yield (ear-basis), and grain yield (plot-basis) for the temperate germplasm (A-D). Linear regression and Pearson correlation for kernel weight, kernels per ear, grain yield (ear-basis), and grain yield (plot-basis) for the tropical germplasm (E-H).

**Supplemental Figure 2.** 30-year month average maximum (red) and minimum (blue) air overlaid on a barplot displaying 14-year monthly precipitation (mm). Lines and bars represent the 14-year average with error bars showing variation among years based on the standard deviation. Triangles and squares represent data from 2017 and 2018, respectively.

**Supplemental Figure 3.** Correlation plot represents phenotypic Pearson correlation coefficients of 25 ear photometry (EP) traits ordered based on first principal component. The value for the correlation is given as a percent.


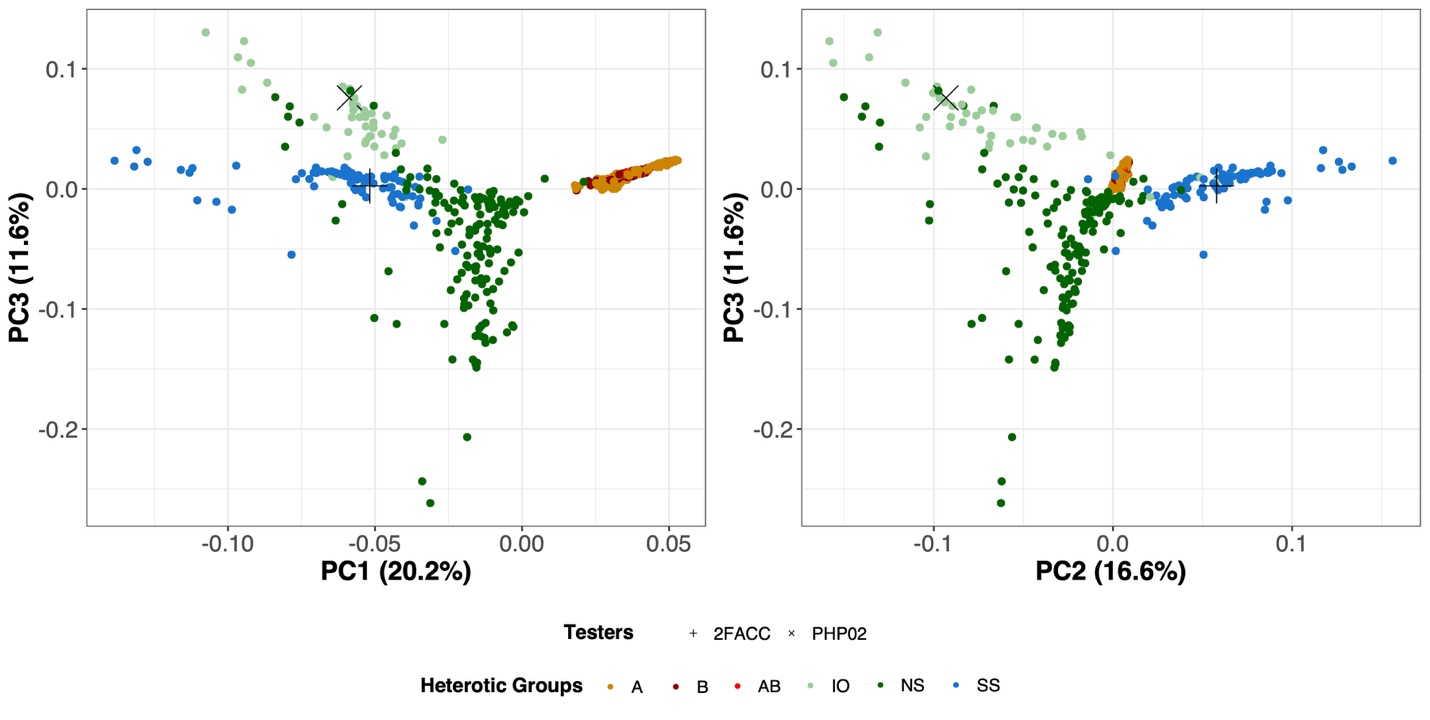


**Supplemental Figure 4.** Principal component analysis (PCA) for 531 inbred lines in this study using 755,339 SNPs. PC1 (x-axis) explains 20.2% of the variation of the SNP data while PC3 (y-axis) explains 11.6% of the variation of the SNP data (Left). PC2 (x-axis) explains 16.6% of the variation of the SNP data while PC3 (y-axis) explains 11.6% of the variation of the SNP data. Testers in this experiment (2FACC and PHP02) are individually labeled with distinct shapes while with heterotic groups are differentiated based on color.

**Supplemental Figure 5.** Principal component analysis (PCA) for 286 inbred lines in this study using 755,339 SNPs. The amount of the variation explained by the principal component is given on the axis label. Color distinguishes the temperate heterotic groups (left). Principal component analysis (PCA) for 245 inbred lines in this study using 755,339 SNPs. The amount of the variation explained by the principal component is given on the axis label. Color distinguishes the tropical heterotic groups (right).
